# Methodology to Evaluate Dripper Sensitivity to Clogging due to Solid Particles: An Assessment

**DOI:** 10.1155/2018/7697458

**Published:** 2018-10-23

**Authors:** Rogério Lavanholi, Fabricio C. Oliveira, Antonio P. de Camargo, José A. Frizzone, Bruno Molle, Nassim Ait-Mouheb, Séverine Tomas

**Affiliations:** ^1^Biosystems Engineering Department, College of Agriculture “Luiz de Queiroz” (ESALQ), University of São Paulo (USP), Piracicaba, SP, Brazil; ^2^Faculty of Agrarian Science (FCA), Federal University of Great Dourados (UFGD), Dourados, MS, Brazil; ^3^College of Agricultural Engineering (FEAGRI), State University of Campinas (UNICAMP), Campinas, SP, Brazil; ^4^National Research Institute of Science and Technology for Environment and Agriculture (IRSTEA), Montpellier, France

## Abstract

Emitter clogging is a major problem in microirrigation systems, which may result from the isolated or combined effects of physical, chemical, and biological agents. Clogging caused by suspended solid particles is the most common plugging form of emitters. Water quality and emitter geometry are key aspects in clogging processes. Any suitable test procedure to assess the sensitivity of drippers to clogging should take into account the predominant factors that influence clogging and must reproduce the field conditions. This research set out to assess the performance and suitability of a laboratory clogging test procedure in order to validate a methodology and to provide scientific results that may support the standardization of a clogging test method. The evaluated methodology has been used by the IRSTEA laboratory since 1974 (Platform of Research and experiment on Science and Technology for Irrigation - PReSTI, formerly LERMI) and its contents are currently being discussed by the ISO TC23/SC18 committee. The aim is to define a standardized testing protocol to evaluate the sensitivity of emitters to clogging due to solid particles. Replications analyzing the clogging resistance of four models of emitting pipes were carried out in a laboratory. The clogging test procedure enabled an accurate assessment of the combinations of concentration and size of particles that caused clogging in each model of dripper. However, a significant variability in degree of clogging was identified when the results of replications for each model of dripper were compared. Several requirements, concerns, and improvements related to the clogging test protocol were discussed.

## 1. Introduction

Clogging of emitters is a serious problem affecting microirrigation systems [1, 2]. The clogging process is influenced by water quality [3] and emitter geometry [4, 5]. In terms of water quality, problems arise from individual or combined effects related to physical, chemical, and biological agents [1, 3, 6]. In the case of surface water sources, clogging caused by suspended solid particles is the most common plugging form of emitters [3, 5] due to particles generally too small to be retained by the filter that aggregate downstream from the filtration system [7].

Methodologies to evaluate or compare the resistance of emitters to clogging may be useful to select the appropriate emitter design for a given set of water characteristics and further recommend a consistent filtration method and grade once the emitters' performance is known. Understanding some of the clogging processes is essential to defining a set of testing protocols required to compare or assess emitter sensitivity to clogging. A testing procedure must include the predominant factors that influence clogging while accelerating the drippers ageing process. The facilities and methodologies to be used must provide results of high repeatability and reproducibility in order to ensure that a given material presents similar results when evaluated by different laboratories.

This research set out to assess a methodology developed by the laboratory of IRSTEA-PReSTI (France) since 1974. Its contents are currently being discussed under ISO TC23/SC18-Irrigation techniques (document ISO DTR 21540), in order to define a standardized testing protocol to evaluate emitter sensitivity to clogging due to solid/mineral particles.

## 2. Materials and Methods

### 2.1. Evaluated Drippers

Experiments were carried out at the Irrigation Laboratory of Luiz de Queiroz College of Agriculture (ESALQ-USP), Piracicaba, SP, Brazil. Four models of emitting pipes accounting for nonpressure compensating flat drippers were tested under a steady 98.1 kPa pressure (Table 1). The emitters were spaced every 0.3 m and integrated to 17 mm nominal diameter polyethylene pipes. Two geometric labyrinth designs were evaluated (A and B). For each design, we picked up a low discharge model (1) and the other of intermediate discharge (2) to analyze the effect of flow rate on clogging sensitivity. Since the key objective of this research was to evaluate a clogging test protocol, the manufacturers and brands of the evaluated drippers are not presented. For drippers identified as B, the only difference between the two models was the labyrinth depth. The coefficient of variation of flow rate (CVq) was obtained experimentally based on the evaluation of 26 emitters of each model. These tests were carried out with new emitters and distilled water.

### 2.2. Testing Facility

**Figure 1:**
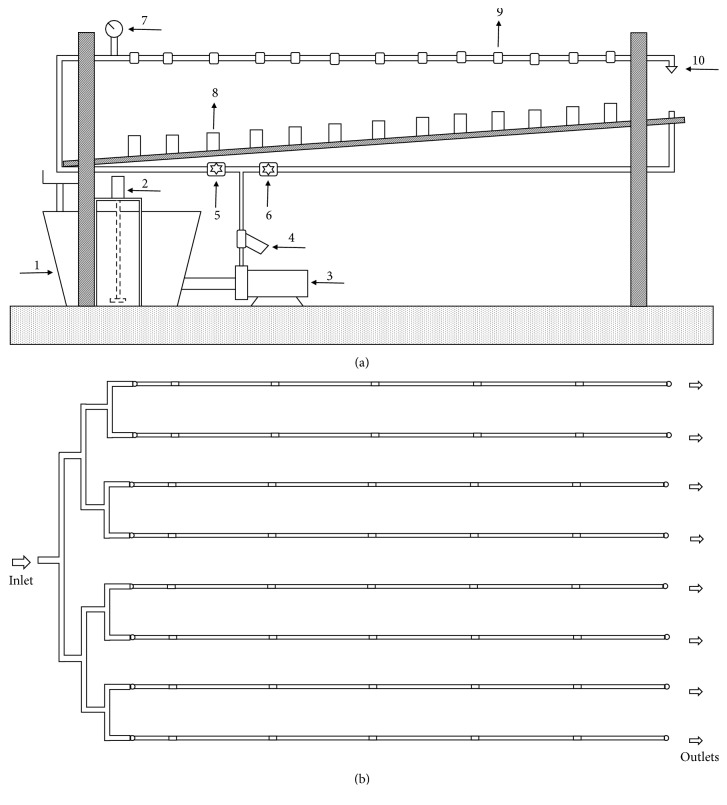
Experimental set-up. (a) Lateral view of the testing bench: (1) water tank; (2) mixer; (3) cast iron pump; (4) 595-*μ*m screen filter; (5) gate valve used to set the testing pressure; (6) gate valve used to set the flow at the gutter upper part; (7) manometer; (8) collector; (9) dripper; (10) sprinkler nozzle. (b) Top view of the testing bench.

The experiments were undertaken at the testing bench shown in Figure 1 designed as a closed circuit in which water circulates during the experiments. A plastic water tank with a volume of 250L and a mixer were used to maintain the solid particles in suspension. The conical frustum shape of the tank helps avoid deposition of particles at the reservoir bottom corners. The mixer has a propeller whose rotation speed is controlled by an electric motor connected to a variable frequency drive. The mixer is coupled to an electric motor of 0.25 CV operating at 354 rpm. The diameter of the mixer's helix is 18 cm and it spaced 11.5 cm from the bottom of the tank. A pump (power = 3 CV, maximum flow rate = 15 m^3^ h^−1^, and maximum pressure head = 46 m) injects water and suspended particles from the bottom of the tank to a manifold, which consists of symmetric bifurcations connected to 8 parallel emitting pipe segments of 5 m length (Figure 1(b)). The pipe that conveys water from the pump to the bifurcated manifold was designed to operate with flow velocities higher than 1 m s^−1^. This velocity ensures that the hydrodynamic force produced is sufficient to transport particles from the tank to the lines, since the critical velocity of deposition for the water distribution system set-up is about 0.6 m s^−1^ obtained by the Durand and Condolios equation [8]. The flow velocity of laterals was fixed to approximately 1 m s^−1^ by installing calibrated nozzles at each lateral outlet. The water from drippers and nozzles flows into a gutter (3% slope) that conveys water back to the tank. The flow velocity into the gutter was relatively high and ensured that all particles were dragged back to the tank. Finally, a screen filter with size equal to 595 *μ*m was installed at the pump outlet in order to trap contaminant debris larger than those added to the tank.

### 2.3. Soil Mixture and Water Quality

The soil mixture employed during the clogging tests was prepared based on requirements defined by [9]. The mixture was prepared from a natural soil obtained by field sampling. The natural soil accounted for 60.6% of clay, 12.5% of silt, and 26.9% of sand. Soil pH was measured on a suspension of soil and deionized water whose ratio was 1:2.5 by volume [10]. A Digimed DM-23 pH meter and an EC meter Digimed DM-32 were used during the experiments. The natural soil pH was 5.70 and its electrical conductivity was 2416 *μ*S cm^−1^.

The soil mixture preparation consisted of dry sieving, destruction of organic matter with oxygen peroxide, dispersion, and wet sieving [9]. A set of 15 sieves were used to obtain the ranges of particle sizes to undertake the experiments. The amount of soil taken from each sieve was calculated to obtain a homogeneous particle size distribution which can be reproduced elsewhere. The pH and electrical conductivity of soil mixture ranges after the whole process of preparation were as follows: (Stage 1) pH=7.77, EC=2510 *μ*S cm^−1^; (Stage 2) pH=9.32, EC=113.4 *μ*S cm^−1^; (Stage 3) pH=9.36, EC=107.8 *μ*S cm^−1^; (Stage 4) pH=9.69, EC=213.1 *μ*S cm^−1^ (Table 2). The increase in soil pH when the natural soil and the soil mixture were compared was due to addition of bases, specially sodium hydroxide, to the soil during the procedure to obtain the mixture of solids particles used as clogging material [9].

The water used to undertake the clogging tests was distilled to ensure reproducibility and repeatability and to reduce side effect such as chemical precipitation that could influence clogging test results.

### 2.4. Clogging Test Protocol

Before each run, the entire testing bench was cleaned and disinfected using a solution containing 2 ppm of free chlorine, whose chemical source was sodium hypochlorite (NaOCl). The disinfection procedure aimed to eliminate or reduce the microorganism population that could promote biofilm growth at the initial stages of clogging tests. Secondly, the distilled water was added to the tank and the initial discharge of emitters was measured. Finally, solid particles corresponding to each testing level were added to the tank.

The clogging test procedure established by IRSTEA consists of 4 stages varying in the size and concentration of particles added to the water tank (Table 2). The particle sizes employed during the experiments were a little different from IRSTEA due to the standard sizes of sieves available in Brazil (2). The test operated 8 h per day and 5 days per week. Each testing stage lasted 40 h and the whole testing procedure lasted 160 h.

The discharge from 26 drippers of each model was measured every day after 8 h of operation. A precision scale (expanded uncertainty = 0.1 g, resolution = 0.01 g) and a stopwatch were used to determine the flow rate of emitters by weighing the amount of water collected during 30 min. The water temperature and water pH were measured daily using a mercury thermometer and a pH meter, respectively.

### 2.5. Outlines of Experiments

Three replications were undertaken under similar testing conditions. At the beginning of each replication, the testing bench was cleaned and disinfected (see previous section), and the emitting pipes were replaced with new ones.

Emitter sensitivity to clogging was evaluated based on the discharge decrease of emitters during the tests. The emitters were considered clogged when their discharge decreased by more than 25% compared with the initial discharge [11–13]. For each model of emitter, the severity of clogging also was quantified by an index called the degree of clogging, *D*_*C*_ (1), which expresses the discharge decrease of a group of emitters due to clogging effects. *D*_*C*_ is zero when a group of emitters is operating normally (i.e., no clog). If *D*_*C*_ is higher than 25%, the group of emitters is considered to be clogged.

(1) $$\begin{array}{c} D_{C}=100\left(\;1-\frac{\sum_{i=1}^{n}q_{i}}{n{\overset{-}{q}}_{i}}\;\right) \end{array}$$

*D*_*C*_ is the degree of clogging (%); *q*_*i*_ is the current discharge of emitter *i* (L h^−1^); *n* is the number of emitters whose discharge has been monitored (dimensionless); ${\overset{-}{q}}_{i}$ is the average flow rate of new emitters free of clogging (L h^−1^) operated under the testing pressure.

Images of clogged emitters were obtained using a Leica M125C stereo microscope. At the end of each replication, emitters were manually opened using a scalpel. Causes of clogging were suggested based on the visual inspection of the labyrinths.

## 3. Results and Discussion

### 3.1. Flow Rate Dynamics and Sensitivity of Emitters to Clogging

**Figure 2:**
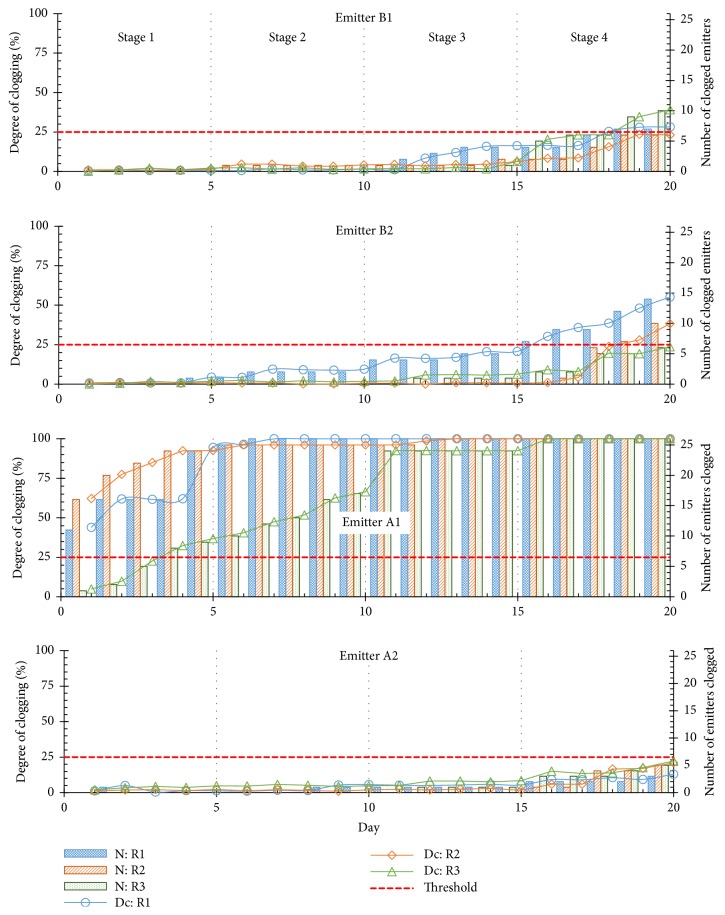
Degree of clogging and number of clogged emitters during the experiments accounting for 3 replications (R1, R2, and R3); N is the number of clogged emitters (i.e., flow rate decreased by more than 25% from the initial value); Dc represents the degree of clogging (1). The threshold represents the level at which clogging is considered to occur.

The results obtained during the experiments are shown in Figure 2. The models B1 and B2 presented a similar clogging resistance with similar trends on the 3 replications. Both models clogged in the last stage of the test; hence they were sensitive to clogging when the concentration of particles was higher than 375 ppm and particle sizes were larger than 212 *μ*m. The only geometrical difference between both models was the labyrinth depth (a difference of 0.1 mm) and it did not influence dripper sensitivity to clogging. Previous studies had found that clogging resistance capability is more influenced by the geometric design of the flow path than by the cross-sectional area or flow rate of emitters [14].

The model A2 did not clog in any of the testing stages and presented the best clogging resistance among the evaluated models of drippers. On the last day of the experiment, the discharge of only 3 to 5 drippers had deviated by more than 25% from the initial value (Figure 2). The model A2 was therefore considered not sensitive to clogging when operated with water containing solid particles of up to 500-*μ*m size and 500-ppm concentration. Although that model presents a simpler labyrinth design when compared to the design of B1 and B2, the filtration grid at the flow path inlet and/or the design of the flow path of the model A2 resulted in an efficient mechanism to prevent blockage of drippers. In addition, A2 has the largest dimensions of flow path among the evaluated models of drippers (Table 1). Specifically for rectangular section channels, the width and depth of the labyrinth channels were reported to enhance clogging resistance performance [15].

On the other hand, the model A1 demonstrated poor clogging resistance performance and was very sensitive to clogging due to solid particles. The drippers were considered clogged in the first stage of the test, in which water accounted for particles of up to 75-*μ*m size at 125-ppm concentration. The clogging hazard due to physical particles is considered severe when water presents a concentration of suspended particles higher than 100 ppm [3] or 400 ppm [16]. A rough comparison between the labyrinth dimensions of A1 and A2 (Table 1) indicates that the dimensions of the A1 are approximately 35% smaller than the other model. The smallest dimension of model A1 is about 0.42 mm (Table 1). Since models A1 and A2 follow the same design, the smaller dimensions of the A1 may explain its poor clogging resistance performance.

**Figure 3:**
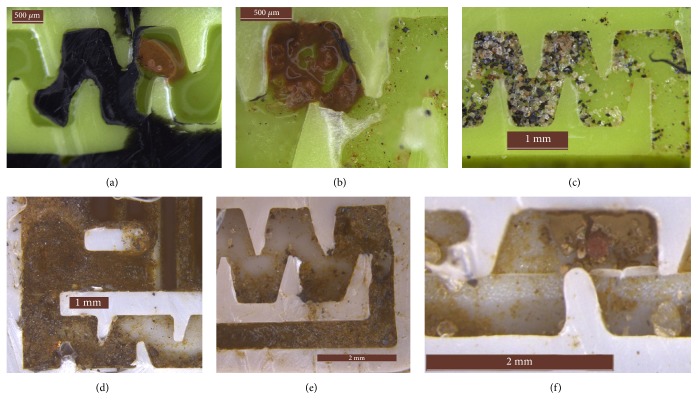
Blockage of the flow path due to the aggregation of fine particles drippers A1 ((a) and (b)). Deposition of big particles within drippers A1 (c). Deposition of particles at the labyrinth inlet and at channel corners for drippers B2 ((d) and (e)). Deposition of particles within B2 due to the molding imperfections of drippers (f).

In Figure 3, images taken at the end of replications from labyrinths of drippers A1 indicated two different behaviors of clogging in this model of dripper. One of the clogging behaviors was due to the aggregation of fine particles (i.e., clay and silt) (Figures 3(a) and 3(b)) and was observed only in the labyrinths of drippers A1. The images indicated the presence of a single aggregate of fine particles formed within the labyrinth and resulting in a total plugging of the dripper. During the experiments, approximately 30% of clogging causes of drippers A1 were associated with the aggregation of fine particles. Although clay and silt particles are much smaller than the labyrinth flow path, under certain hydrodynamic and physicochemical conditions, the fine particles may aggregate into larger particles and are not fragmented by shear stress [7]. These aggregates are able to block the whole flow path (Figures 3(a) and 3(b)). The electric charges on the particle surface influence the aggregation potential and may be quantified by an index known as Zeta Potential (*ζ*), which reflects particle nature and is diversely influenced by pH and concentration of salts in the aqueous media [7, 17]. As the value of *ζ* deviates from zero, the potential for aggregation becomes lower [18].

The other clogging behavior is related to the accumulation of relatively large particles (i.e., fine sand) at the labyrinths' inlet and at channel corners (Figure 3(c)). The deposition of particles in corners is related to the recirculation zones and low velocity that promotes the trapping of particles [19, 20]. In addition, the successive cycles of operation every 8 h allow particles to deposit in the pipes and labyrinths when the system is turned off. Thereafter, when the system is restarted again, the flow is not enough to flush all the particles from the vortex zones to the emitter's outlet.

Clogging due to the accumulation of relatively large particles at the labyrinth inlet and at channel corners is also observed in the other models of drippers investigated during the experiments and seems to be the most common type of plugging. Figures 3(d) and 3(e) show pictures taken from drippers B2 at the end of the test, indicating an accumulation of particles at the entrance of the labyrinths that did not result in significant discharge drop in all drippers.

Finally, drippers B1 and B2 presented barbs across the flow path resulting from molding imperfections (Figure 3(f)). The deposition of particles upstream of such barbs was consistently observed, since those imperfections blocked part of the labyrinth flow section promoting clogging. This highlights the strategic importance of production accuracy that does not systematically result in an observable manufacturing variability as expressed in the coefficient of variation (CVq) defined in [21].

### 3.2. Assessment of the Clogging Test Procedure

In each replication, four dripper models were evaluated simultaneously under the same testing conditions. The clogging resistance performance varied according to the model of dripper. The results are consistent with the literature and clearly indicate that emitter design is a key aspect in terms of clogging resistance performance [4, 5, 13, 14, 22, 23].

Comparing the results from three replications, each dripper model always clogged at the same test stage (Figure 2). This indicates that the clogging test procedure enabled a correct assessment of which combination of concentration and particle size caused clogging in each model of dripper.

Regarding the results of drippers A2 (Figure 2), the series of lines corresponding to the three replications are close, and the repeatability can therefore be considered reasonable, in a qualitative way. However, analyzing the other models, significant variability in degree of clogging (*D*_*C*_) was identified when comparing the results of replications within each model. On a daily timescale, the highest deviations between maximum and minimum values of *D*_*C*_ were 16.0, 31.7, and 67.6% for the corresponding models B1, B2, and A1 (Figure 2). No technical reference currently states what would be an allowed variation of results among replications, nor does any methodology systematically quantify the variability among replications of such experiments; it is therefore difficult to affirm whether the variability in *D*_*C*_ was excessive or not. Anyway, the variability of results seems to be excessive for the other models, especially for A1. As we have seen, for each replication, all the models were tested simultaneously under the same testing conditions and thus the variability among replications might be explained by noncontrolled variables that influenced each model of dripper differently. Although the clogging test protocol was developed only to identify trends in terms of clogging sensitivity due to solid particles, a minimum degree of similitude will be obtained in the results when the test is repeated under similar testing conditions. While such a level of result repeatability is not satisfactory, it is necessary to find out what in the test protocol induces such variations.

Drippers A2 did not show significant variability among replications probably because this model was highly resistant to clogging under the evaluated conditions; hence noncontrolled variables that might interfere in the flow rate of other models of drippers do not influence discharge of A2 drippers.

The water pH at the beginning of the tests was about 7 and consistently increased to approximately 8 immediately after the particles of Stage 1 (Table 2) were added to the tank due to the chemical properties of the particle mixture. Following that, water pH fluctuations of ±0.2 occurred during the test. Although the initial water temperature ranged from 22 to 24°C, the continuous water circulation in the testing bench naturally increased the water temperature to an upper value in which the system reached a quasi-steady condition. Under such conditions, the water temperature ranged from 26 to 30°C and the fluctuations were related to normally occurring daily temperature variations.

### 3.3. Highlights, Concerns, and Recommendations Related to Clogging Tests

Based on the experiments, some highlights, concerns, and recommendations related to clogging tests may be helpful before further activities are undertaken.

As the test aims to evaluate emitter sensitivity to clogging due to solid particles, microorganism growth should be avoided in order to prevent interactions among physical and biological agents of clogging (e.g., the presence of biofilm is strongly suspected in the clogging material found in some of the drippers evaluated, though tests were not conducted to confirm this). Chlorine is widely used for disinfecting water due to its stability and effectiveness [24]. Continuous chlorination in the tank would be interesting to prevent biological interferences in the tests.

The influence of chemical agents of clogging should also be avoided considering that the clogging test procedure aims to evaluate sensitivity to solid particles. Although distilled water was used during the experiments, a significant number of chemical elements might have been solubilized from the soil compound, which was added to the water. The precipitation of ions is affected by several factors, although water pH seems to be the most important. When water pH is maintained lower than 7, most clogging problems related to precipitation of ions are mitigated [25]. An adjustment of water pH around 6.5 (+/- 0.5) should therefore be part of the clogging test protocol in order to prevent chemical precipitation during tests.

Another important aspect around the proposed clogging test protocol relates to the mixture of particles. The first concern is related to the solid shape and dynamics of these particles when transported by fluid flow. Suspended particles in irrigation waters are of various shapes and present smooth edges because of wearing processes occurring in the nature. This is the reason why particles are sieved from in-field samples and may present some variability. Standardized commercial compounds could be used to prepare the mixture of particles, but such compounds account for sharp particle edges due to their production process (i.e., crushing of big aggregates of soil to obtain an expected range of sizes) and usually have higher density than natural particles. The flow dynamics of spherical or smooth-angled shape suspended particles within pipes or labyrinths are quite different from sharp-angled particles. The second concern refers to the mineralogy and chemical composition of soil, since both characteristics may influence particle aggregation phenomena. Probably the nature of clay particles added to the soil compound should be standardized, since aggregation potential relies upon the nature of particles [7, 17]. Finally, the soil preparation based on [9] is time-consuming, which could be avoided in case of using standardized commercial compounds. The trade-off between using a standardized commercial compound or a soil mixture prepared from in-field soil samples requires further investigation.

The clogging test procedure does not allow for separation between clogging caused by concentration and the size of particles. When a test progresses from one stage to the next, the previous water mixture remains in the tank and larger particles are added to the mixture. Moreover, samples of emitting pipes are not replaced from one stage to the next; there is therefore a residual effect that prevents us from differentiating between clogging caused by the concentration and size of particles. The protocol considers that clogging severity is more influenced by particles size range than concentration because when drippers are not clogged at a given stage, they are not considered to be sensitive to the size of particles used at that stage. The idea that clogging rate is more affected by particle size than concentration is also supported by previous research [4]. Assuming that clogging is mainly influenced by particle size, one practical recommendation consists of adding a sieve or filter able to trap particles larger than those required to avoid emitter clogging. On the other hand, the idea that concentration of particles is highly significant in clogging severity is also supported by a previous study which observed that the filtration level of screen filters is determined as 1/7 – 1/10 of the labyrinth's channel width [15]. The same study also reported that sand particles larger than 1/5 of the channel width plugged emitters, while particles between 1/5 and 1/7 of the channel width caused a partial clogging of drippers during evaluations. Such comments indicate that both the concentration and size of particles are important while selecting the filtration system for microirrigation, but ultimately the subject is not clearly addressed in the literature.

Finally, the use of pumps whose impeller and housing are made of cast iron is not recommended. Depending on water and solid particles characteristics, iron particles may detach from inner parts of the pump due to corrosion or abrasion. Such particles may escape filtration and reach the emitters resulting in their clogging or the development of ferro-bacteria biofilm. The use of pumps consisting of impeller and housing made of stainless steel is strongly advised for further researches. In addition, a methodology for real time monitoring of the concentration of particles within the tank or in the pipes would be helpful to monitor the testing conditions.

Finally, the number of drippers that should be monitored in a clogging test protocol in order to improve result reliability remains unanswered.

## 4. Conclusions

The clogging test procedure enabled an accurate assessment of the combinations of concentration and size of particles that caused clogging in each model of dripper.

A significant variability in degree of clogging was identified when comparing the results of replications for one model of dripper. The excessive variability might be explained by noncontrolled variables that influenced each model of dripper differently.

The experiments demonstrated that the results of clogging tests are quite sensitive and may change significantly due to minor details related to the methodology or testing facilities. Additional requirements and details related to the clogging test protocol and its facilities must be more accurately defined to ensure the repeatability and reproducibility of results.

Recalling that the objective of such test is to give trends in sensitivity to clogging of one dripper model, due to its design and its manufacturing quality that may not be revealed by standard hydraulic test (ISO 9261), research is still needed to refine understanding of the influence (or not) of some parameters: pH, conductivity, and particles zeta potential.

## Figures and Tables

**Table 1:** Characteristics of the evaluated drippers.

Emitting-pipe identification	A2	A1	B2	B1
Nominal discharge [L h^−1^]	1.7	0.6	1.6	1.2

Average discharge under 98.1 kPa [L h^−1^]	1.55	0.55	1.39	1.15

Coefficient of variation - CVq [%]	1.7	2.0	2.2	2.3

Design	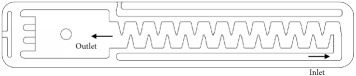		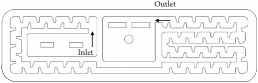	

Labyrinth dimensions [top view / units=mm]	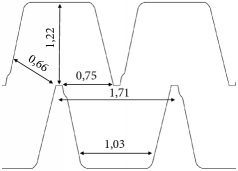	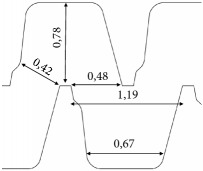	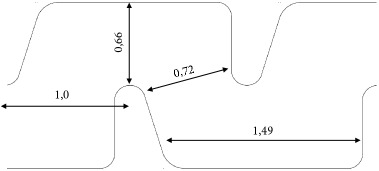	

Labyrinth depth [mm]	0.71	0.42	0.64	0.54

Internal diameter of the pipe [mm]	16.0	16.0	15.5	15.5

**Table 2:** Size and concentration of particles in each testing stage.

Stage	Duration [h]	Range of particles size*∗*			
		< 75 *μ*m	75 – 125 *μ*m	125 – 212 *μ*m	212-500 *μ*m
		Concentration of particles [mg L^−1^]			
1	0 – 40	125	-	-	-
2	40 – 80	125	125	-	-
3	80 – 120	125	125	125	-
4	120 – 160	125	125	125	125

*∗* Ranges of particle sizes adopted by PReSTI/IRSTEA: Stage 1: <80 *μ*m; Stage 2: 80-100 *μ*m; Stage 3: 100-200 *μ*m; Stage 4: 200-500 *μ*m.

## Data Availability

The data used to support the findings of this study are included within the article and any additional data can be available upon request from the authors.
